# CTLA-4 blockade during dendritic cell based booster vaccination influences dendritic cell survival and CTL expansion

**DOI:** 10.1186/1476-8518-5-9

**Published:** 2007-07-29

**Authors:** Anders E Pedersen, Franca Ronchese

**Affiliations:** 1 Department of International Health, Immunology and Microbiology, The Panum Institute, University of Copenhagen, Denmark; 2 Malaghan Institute of Medical Research, Wellington, New Zealand

## Abstract

Dendritic cells (DCs) are potent antigen-presenting cells and critical for the priming of CD8+ T cells. Therefore the use of these cells as adjuvant cells has been tested in a large number of experimental and clinical vaccination studies, in particular cancer vaccine studies. A number of protocols are emerging that combine vaccination with CTL expanding strategies, such as e.g. blockade of CTLA-4 signalling. On the other hand, the lifespan and *in vivo *survival of therapeutic DCs have only been addressed in a few studies, although this is of importance for the kinetics of CTL induction during vaccination. We have previously reported that DCs loaded with specific antigens are eliminated by antigen specific CTLs *in vivo *and that this elimination affects the potential for *in vivo *CTL generation. We now show that CTLA-4 blockade increases the number of DC vaccine induced LCMV gp33 specific CTLs and the lysis of relevant *in vivo *targets. However, the CTLA-4 blockage dependent expansion of CTLs also affect DC survival during booster DC injections and our data suggest that during a booster DC vaccine, the largest increase in CTL levels is already obtained during the first vaccination.

## Background

Dendritic cells are sentinel cells in the peripheral tissues. After exposure to inflammatory cytokines together with pathogen associated molecular patterns they undergo maturation, migrate to the regional lymph nodes and initiate CD4+ and CD8+ T cells responses [1-3]. In particular the potent priming of CD8+ T cells into CTLs with the capacity for recognition and killing of target cells has attracted much attention in cancer vaccination protocols [4,5].

A number of strategies have been identified for the expansion of CTL's such as PD-1 ligand blockade [6], agonistic 4-1BB monoclonal antibody [7] and CTLA-4 blockade. CTLA-4 normally competes with CD28 for CD80 and CD86 binding and thereby acts as a negative regulator of T cell activation [8]. In addition CTLA-4 is expressed by CD4+CD25+ natural occurring regulatory T cells which in this way inhibit DC function and bystander T cells [9-11]. CTLA-4 blockade is therefore a potent strategy for the amplification of immune responses against weak antigens, e.g. tumour antigens, during vaccination [12-14] and is currently being tested in clinical cancer trials [15,16].

The survival of injected DC is of critically importance for the *in vivo *induction of CTLs during DC based vaccination. We have previously shown that in antigen primed mice, injected DCs are eliminated before they reach the draining lymph node (DLN) and their interaction with memory or naïve T cells is therefore limited at this site. This elimination is performed by activated CD8+ T cells and is dependent on perforin secreted from these cells [17,18]. Under normal physiological conditions this probably acts as a feedback mechanism to prevent exaggerated expansion of CD8+ T cells during a viral infection [19,20], but the phenomenon might at the same time limit the potential of DC based vaccines in therapeutic settings [18,21].

We now show that CTLA-4 blockade increases the number of DC vaccine induced CTLs and the lysis of *in vivo *target cells. However, antigen-loaded DCs are eliminated after repeated injection in primed animals and the CTLA-4 blockage dependent expansion of CTLs leads to a decrease in surviving DC reaching the lymph node after a second DC injection. Our data suggest that repeated DC vaccine combined with e.g. CTLA-4 blockade does not increase the CTL expansion over time due to elimination of injected DCs in a primed host whereas CTLA-4 blockade provide a potent increase in CTL numbers when delivered together with the primary DC vaccination.

## Methods

### Mice

Conventional 6–8 week old female C57Bl/6 mice were purchased from Taconic Europe (Ry, Denmark) and kept under controlled microbial conditions at the local animal facility.

### Generation of BM-DC

DCs were generated from BM cells derived from C57Bl/6 mice. BM-cells from femurs and tibias were washed and cultured overnight in 6-well plates (TPP, Trasadingen, Schwitzerland) at 2 × 10^6 ^cells/ml in 3 ml culture medium/well. Culture medium (CM) was RPMI-1640 with Glutamax supplemented with 10% FCS (Harlan Sera-Lab Ltd, Hillcrest, England) and antibiotics. The next day, non-adherent cells were harvested and resuspended in CM containing 10 ng/ml GM-CSF plus 20 ng/ml IL-4 (both from Peprotech, Rocky Hill, NJ, USA) and cultured at 1 × 10^6 ^cells/ml in 3 ml CM/well. Fresh cytokines and medium were added on day 3. Day 6 DCs were harvested as non-adherent and loosely adherent cells. These cells have previously been described to be 60–90 % CD11c positive cells with DC characteristics [22].

### Immunization with BM-DC and CTLA-4 blockade

Day 6 DC were harvested and incubated with 40 μM of the H-2 D^b^binding 33–41 fragment of LCMV glycoprotein gp33 KAVYNFATM peptide (from Schäfer-N, Copenhagen, Denmark) for 2 hours at 37°C and then administered by subcutaneous injection as 1*10^6 ^cells/mouse. The hybridoma 9H10 which produces monoclonal hamster anti mouse CTLA-4 antibody was kindly provided by Dr. Rienk Offringa and has been described previously [14]. The 9H10 antibody was administered as 100 μg/mouse i.p. on the first day together with DC vaccination and 50 μg/mouse i.p. on the third and fifth day. We and others have previously demonstrated that control hamster antibody is without effect in similar experiments (data not shown) [12-14].

### ELISPOT assay

For the ELISPOT assay splenocytes (5 × 10^6^/well in 2 mL/well in 24-well plates (Invitrogen)) were cultured for 8 days with 10 μM peptide (KAVYNFATM) with addition of 100 IU/mL recombinant human IL-2 (Proleukin, Chiron) at day 1 and then used in the ELISPOT assay. 96-well nitrocellulose plates (Millititer, Millipore, Bedford, MA) were coated with anti-mouse IFN-γ (551216 from BD-Pharmingen) in PBS overnight at room temperature. Then, wells were washed with PBS and blocked with Ultraculture medium (BioWhittaker (BE12-725F), Berkshire, England) for 2 h at 37°C. Titrated numbers of the *ex vivo *restimulated cells, with or without the addition of 10 μM peptide, were incubated for 20 h in the antibody-coated plates at 37°C and 5% CO_2_. Plates were then developed with biotinylated anti-mouse IFN-γ (554410 from BD-Pharmingen) and streptavidin-conjugated peroxidase (Dako, Copenhagen, Denmark) followed by 200 μl of substrate [including 1 tablet 4-chloro-1-naphthol 30 mg (057h8927, Sigma) and 5 μl H_2_O_2 _30% (H1009, Sigma)].

### VITAL assay in vivo

*In vivo *cytotoxicity was assessed on fluorescence labelled syngeneic spleen cell populations administered i.v. into mice in equal proportions. Labelling was performed as described previously [23]. The peptide Ag^- ^targets were labeled with CMTMR (orange fluorescent dye chloromethyl-benzoyl-aminotetramethyl-rhodamine, Molecular Probes), and the peptide Ag^+ ^populations were labeled with CFSE (fluorescent dye carboxyfluorescein succinimidyl ester, Molecular Probes, Eugene, OR), thereby providing discreet populations discernible by FACS. The mixed target cell preparation was injected as 4*10^6 ^cells i.v. into different groups of mice including naïve hosts to assess for skewing of population size at the outset of the experiment. Specific lysis of the Ag^+ ^populations was assessed at 24 h after target cell administration by FACS analysis of blood taken from the lateral tail vein. Ag^-^CMTMR labelled cells were detected in FL-2 and Ag^+^CFSE in FL-1 channel. The percentage of surviving Ag^+^spleen cells in immunized mice could then be calculated on the basis of Ag^- ^cells which were not deleted in immunized mice compared to naïve mice and cytotoxicity was calculated as specific lysis according to the following formula:

%specific lysis = 100 - %adjusted survival

where

adjusted%survival = 100 × (%survival of Ag+ cells/(average % survival of Ag+ cells in naïve mice in the absence of effector cells))

### DC labelling, in vivo transfer and recovery

DC were labeled with CFSE by incubation at 5 × 10^6 ^cells/ml in PBS containing 1 μM CFSE for 10 min at 37°C, followed by one wash in 5 vol of ice-cold PBS and two washes in IMDM and loaded with KAVYNFATM peptide. Another fraction of DC were labeled with CMTMR by incubation at 5 × 10^6 ^cells/ml in pre-warmed CM supplemented with 10 μM CMTMR at 37°C for 15 min, followed by incubation in CM alone for a further 20 min as published previously [17].

Mice received 1 × 10^6 ^CFSE-labeled DC loaded with peptide and 1 × 10^6 ^CMTMR-labeled antigen unloaded DC in a total volume of 50 μl IMDM by subcutaneous (s.c.) injections into the distal forelimb (volar aspect). The presence of fluorescent cells in the draining axillary and brachial lymph nodes was then determined after 48 hours. DLN were removed and digested in 2.4 mg/ml collagenase type II (Gibco-Life Technologies) and 1 mg/ml DNAse I (Sigma) for 90 min at 37°C. Lymph node cell suspensions were analyzed using a FACSort (Becton-Dickinson, Mountain View, CA) and CellQuest software (Becton-Dickinson). The region containing DC was identified on the basis of FSC-SSC profile. Data are expressed as the mean percentage of fluorescent cells found within this gate for each experimental group. CTL-mediated elimination of antigen-loaded DC is expressed as a ratio of DC loaded with antigen over DC without antigen. No difference in propidium iodide uptake was observed in harvested DCs from immunized or naïve mice.

### Statistics

Significant differences between sample means were determined with the one-tailed Student's t test for independent samples, and results were considered significant when p < 0.05. Only results presented in the last figure were also significant with a two-tailed Student's t test for independent samples.

## Results

### CTLA-4 blockade increases CTL number and *in vivo *lysis of target cells during DC vaccination

**Figure 1:**
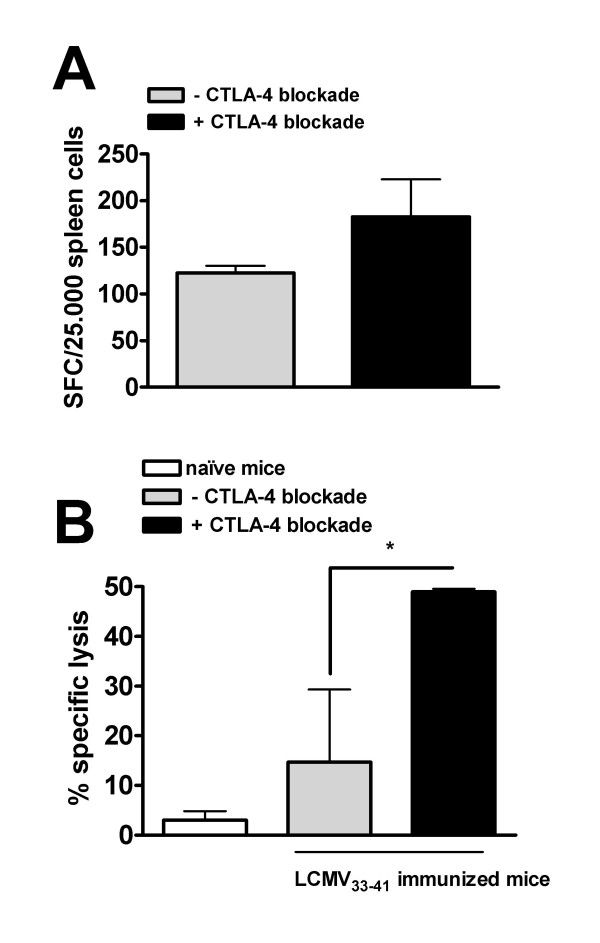
CTLA-4 blockade increases the induction of LCMV gp33_33–41 _specific CTLs and *in vivo *lysis of target cells. (A) C57/Bl6 mice were immunized with peptide LCMV gp33_33–41 _loaded DCs in combination with i.p. injection of anti-CTLA-4 mAb. Spleen cells were isolated 7–10 days after the primary immunization and cocultured with LCMV gp33_33–41 _peptide + IL-2 and then tested for reactivity against the peptide in an IFN-γ ELISPOT assay. (B) Alternatively, peptide LCMV gp33_33–41 _loaded CFSE labeled and peptide unloaded CMTMR labeled spleen cells were injected i.v. in immunized mice and naïve mice and target cell lysis was analyzed after 24 hours by the in vivo VITAL assay. Results are shown as mean ± SD of three mice in 1 representative experiment out of 2. (* p < 0.05)

DC based vaccination is effective for *in vivo *generation of CTL's specific for H-2 D^b ^binding LCMV gp33 derived KAVYNFATM peptide [24] and *in vivo *treatment with anti-CTLA-4 mAb augments the accumulation and activation of adoptively transferred gp33 _33–41 _specific transgenic T cells [25]. We tested the ability of CTLA-4 blockade to expand the number of wildtype CTLs during a single DC vaccination with LCMV gp33_33–41. _As shown in fig 1A, the number of specific CTLs identified in an ELISPOT assay of spleen cells tend to increase, although this was not significant (p = 0.11). We then assessed whether this CTL expansion lead to an increased lysis of target cells *in vivo*. We and others have previously shown that specific killing of fluorescence-labeled peptide loaded syngeneic splenocytes can be used to assess T-cell-mediated cytotoxic activity *in vivo *[23]. Using this assay, cytotoxic capacity of the induced CTLs was assessed 10 days after DC vaccination with LCMV gp33_33–41 _as the % specific lysis of i.v. administered LCMV gp33_33–41 _loaded syngeneic splenocytes. Specific lysis was observed only in the immunized animals, and was significantly increased (p = 0.04) in animals co-treated with CTLA-4 blocking antibody (Fig 1B).

### CTL numbers during repetitive DC vaccination and CTLA-4 blockade

**Figure 2:**
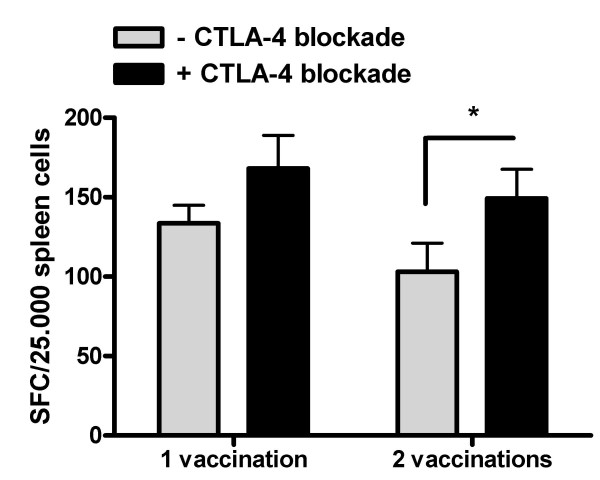
LCMV gp33_33–41 _specific CTL levels are stable during repetitive DC vaccination. C57/Bl6 mice were immunized with peptide LCMV gp33_33–41 _loaded DCs day -14 and -7 in combination with i.p. injection of anti-CTLA-4 mAb. Spleen cells from individual mice were isolated day 0, cocultured with LCMV gp33_33–41 _peptide for 7–10 days and tested for reactivity against the peptide in an IFN-γ ELISPOT assay. Results are shown as mean ± SD of eight mice from two separate experiments. (* p < 0.05)

Repetitive vaccination is a common strategy for boosting of immune responses, by e.g. increasing specific CTL levels. However, the strategy might have potential flaws and limits during DC vaccination. We tested the number of LCMV gp33_33–41 _specific CTLs induced after 1 and 2 vaccinations with LCMV gp33_33–41 _loaded DCs in an IFN-γ ELISPOT assay (Fig 2). To our surprise the number of CTLs was not increased after the second vaccination, but rather exhibited a small non-significant decrease instead. Likewise, two vaccinations combined with CTLA-4 blockade did also not improve CTL expansion (Fig 2) compared to treatment with a single vaccination + CTLA-4 blockade. However, CTLA-4 blockade at the second vaccination significantly increased the number of specific CTLs at the second vaccination (p < 0.05)

### CTLA-4 blockade increase DC elimination during repetitive DC vaccination

**Figure 3:**
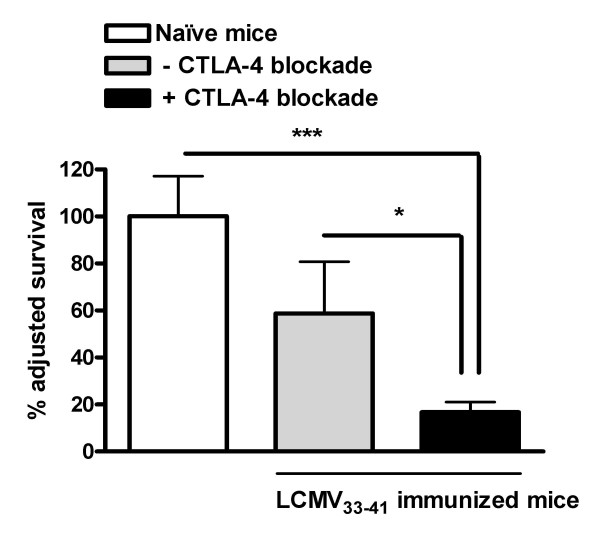
Enhanced DC elimination during DC vaccination combined with CTLA-4 blockade. C57/Bl6 mice were immunized with peptide LCMV gp33_33–41 _loaded DCs with or without i.p. injection of anti-CTLA-4 mAb. After 7–10 days, an inoculum consisting of peptide LCMV _33–41 _loaded CFSE labeled together with peptide unloaded CMTMR labeled DCs was injected subcutaneously into the distal forelimb of naïve mice (control), immunized mice or immunized mice cotreated with anti-CTLA-4 mAb. DCs were recovered from the draining lymph node for FACS analysis and determination of % surviving DCs. Results are shown as mean ± SD of nine mice from 3 separate experiments. (* p < 0.05; ***p < 0.0001)

We next tested the effect of CTLA-4 blockade on DC elimination during a second vaccination. Using a method to directly compare the proportion of antigen-loaded to non-antigen-loaded DC within the same inoculum of cells and in the same host [17] we have shown in previous experiments that in the course of DC based vaccination, DC appearance in the draining lymph node of immunized mice is decreased. A CFSE^+ ^labeled DC population was loaded with LCMV gp33_33–41 _peptide prior to injection, while the non-antigen-loaded CMTMR^+ ^labeled DC population served as a control. The two populations of DCs were then mixed together in equal numbers before injection *in vivo*, so that the numbers of antigen-loaded DCs and non-antigen-loaded DCs could be evaluated within the same recipient lymph node. DCs were then harvested from DLN 48 h later, a time point where DC elimination has previously been shown to be suboptimal [17]. When DCs were administered to animals that were immunized with LCMV gp33_33–41 _loaded DC, only 58 % of the antigen-loaded DC had survived and reached the DLN 48 h later. None of the unloaded DCs were eliminated and no elimination of antigen-loaded DCs was observed in naïve control mice. However, in mice co-treated with CTLA-4 blocking antibody only 17 % of the DC had survived and reached the DLN 48 h post injection (Fig 3). This survival was significantly decreased compared to survival in immunized mice with no CTLA-4 blockade and in naïve mice. We did not identify any difference in surface expression of costimulatory molecules such as CD80 and CD86 on injected DCs from mice treated with CTLA-4 blockade compared to untreated mice (data not shown).

## Discussion

The present study demonstrates that CTLA-4 blockade increases the number of DC vaccine induced LCMV gp33_33–41 _specific CTLs and the lysis of relevant *in vivo *targets. General vaccination approaches take advantage of repetitive vaccinations as a mean to boost the immune response and expand the number of specific CTLs. However, the expansion of CTLs mediated by CTLA-4 blockade also affects DC elimination during repetitive DC injection. Our data suggest that repetitive DC vaccination with or without CTL expanding strategies, e.g. CTLA-4 blockade does not increase CTL expansion compared to the levels obtained after the primary vaccination and that this is due to elimination of injected DCs in a primed host.

Previous reports have documented that CTLA-4 blockade is a feasible strategy for potent *in vivo *expansion of antigen specific T cells, in particular in the context of cancer vaccination [14,15]. Even unspecific expansion elicited by anti-CTLA-4 mAb can be useful both in experimental models and clinical settings [13,15]. Similar, we observed an increase in LCMV gp33_33–41 _specific CTLs and an increased *in vivo *lysis of target cells after LCMV gp33_33–41 _targeting DC based vaccine combined with CTLA-4 blockade. However, since LCMV gp33_33–41 _is already a strong immunodominant epitope, this relative increase is probably smaller compared to relative increases observed for CTLs specific for weaker antigens, such as tumour antigens [14]. This might explain, why *in vivo *tumour prophylactic experiment with DC based vaccination against gp33 positive tumour cells did not clearly show an increased effect of CTLA-4 blockade despite increased CTL levels (data not shown). Also, the level of specific CTLs shown was low as we tested the effect of CTLA-4 blockade after the primary vaccination.

We have previously shown that DC elimination during DC based vaccination is due to the presence of primed antigen specific CTLs and is dependent on perforin expression [17,18]. This phenomenon is likely to limit the potential of DC based booster vaccines in therapeutic settings [18,21]. Indeed, in a number of DC based vaccination studies, in particular in cancer patients, CTL responses are either observed in a low fraction of patients or with great fluctuation and even a decrease in CTL number during vaccination has been reported [5,26,27]. In these early studies, repetitive vaccination with immature or intermediate mature DCs unexposed to potent maturation reagents was used for booster vaccination with the same antigen. Thus, the low fraction of CTLs induced in these studies might be a result of time dependent elimination of injected DCs at booster vaccinations. Unfortunately CTL responses were most often measured after several vaccinations and make it difficult to compare the CTL levels with the levels after first vaccination. In contrast, at least in *in vitro *studies, DC elimination is minimal when LPS matured DCs are applied due to expression of the serpin serine protease inhibitor 6 [28]. In this study, we demonstrate that also the application of CTL expanding strategies such as CTLA-4 blockade lead to a massive loss of surviving DCs during booster vaccination. Since our tumour challenge experiments with addition of CTLA-4 blockade didn't correlate well with CTL levels in an experimental LCMV tumour model, it is unknown if this DC depletion will influence the outcome of a tumour vaccine. Indeed, CTLs might be reactivated during the killing of DCs, and the remaining DC's might be particular potent CTL activators. However, previous studies from our laboratory suggest that the induction of tumour immunity is limited by DC elimination [21]. Therefore, DC elimination, in addition to TH1/TH2 promoting capacities and migration of the DCs to DLN, is an important issue, when designing maturation regimens for DCs used in vaccination studies, in particular in human studies where toll-like receptor ligands such as LPS are not approved for clinical trials. Also, recent research has established that mature DCs are more potent than immature DCs in DC based vaccination studies [3,31] and elimination of immature DCs during vaccination might be one of the reasons.

In conclusion, CTLA-4 blockade dependent expansion of CTLs increases DC elimination during repetitive DC injection and suggests that alternative strategies, such as prime-boost strategies with exclusion of DCs at booster vaccinations [29] or heterologous booster vaccinations [30] designed with alternate epitope loading of DCs during vaccination, should be applied when DC are used for repetitive vaccination with or without inclusion of CTL expanding strategies, such as CTLA-4 blockade.

## Authors' contributions

AEP conceived the study, carried out the in vivo experiments and flowcytometry, performed the statistical analysis and drafted the manuscript. FR participated in the design and coordination of the study and drafted the manuscript. Both authors read and approved the final manuscript.
